# Supplementary material for: STAT2 Promotes Tumor Growth in Colorectal Cancer Independent of Type I IFN Receptor Signaling
Source: Curr Oncol. 2025 Dec 16;32(12):707. doi: 10.3390/curroncol32120707 (PMC12732172; doi:10.3390/curroncol32120707)
Supplement: Supplementary file 1 [file curroncol-32-00707-s001.zip › curroncol-3992821-supplementary.pdf]

Figure S1: Levels of STAT1, STAT2 and STAT3 after deletion of IFNAR1 or STAT2

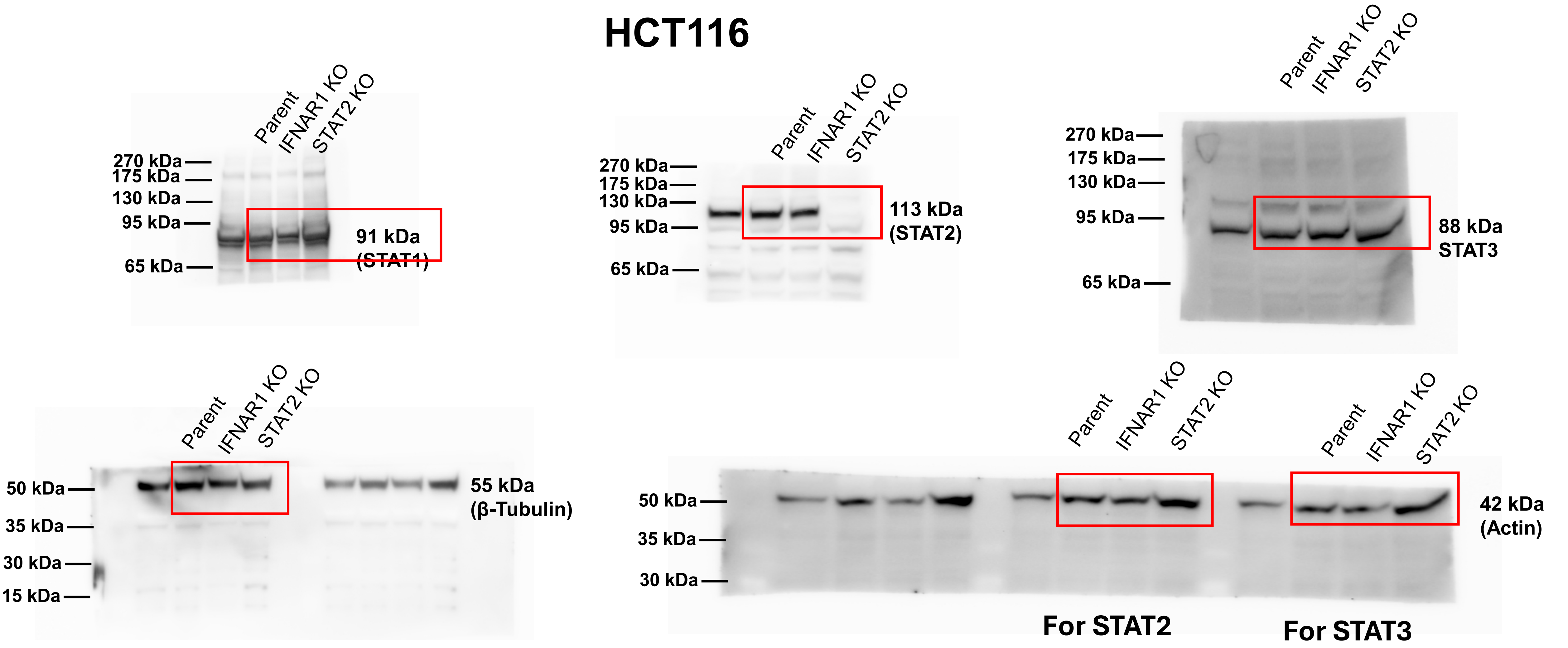

[] : Figure 2a

Figure S2. Activation of STAT1, STAT2 and STAT3 by IFN-α following deletion of IFNAR1 and STAT2

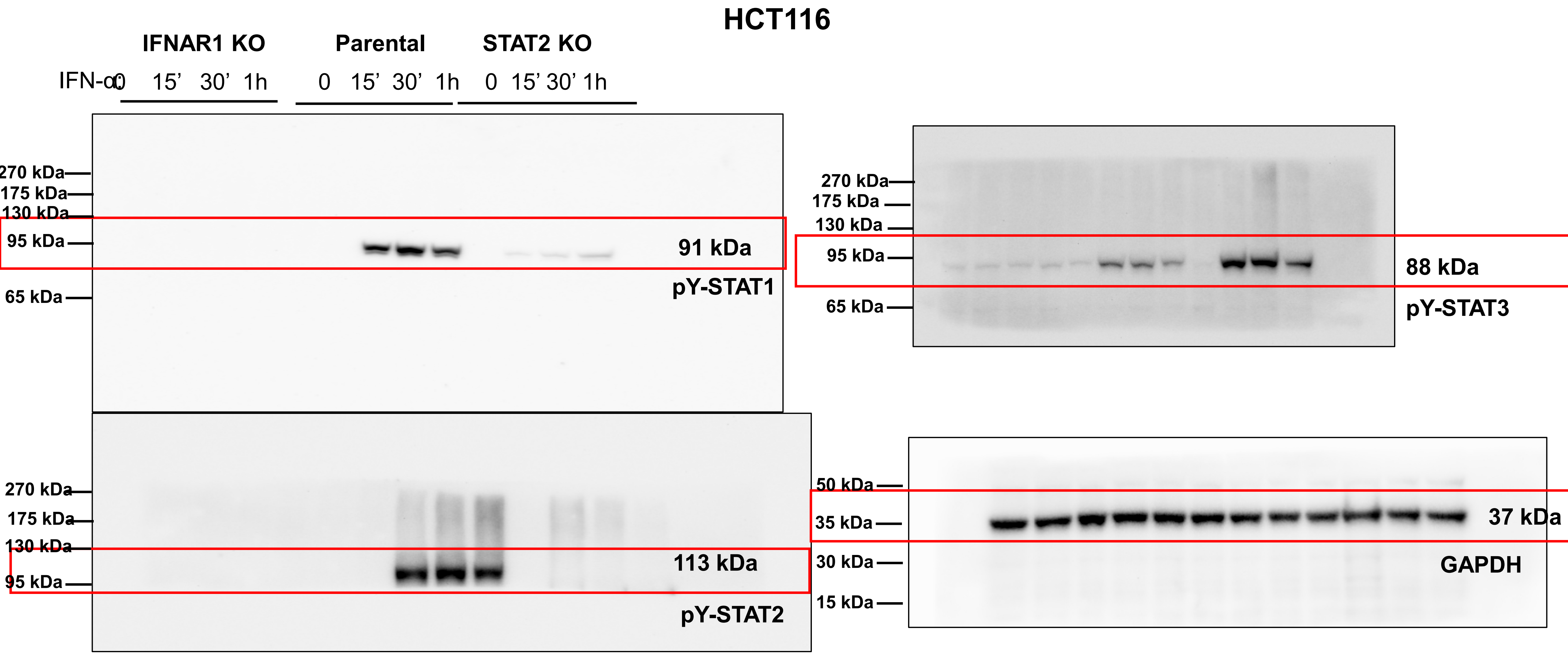

[] : Used in Figure 2b

Figure S3: Activation of STAT1 by IFN-β after deletion of IFNAR1 and STAT2 in MC38 cells

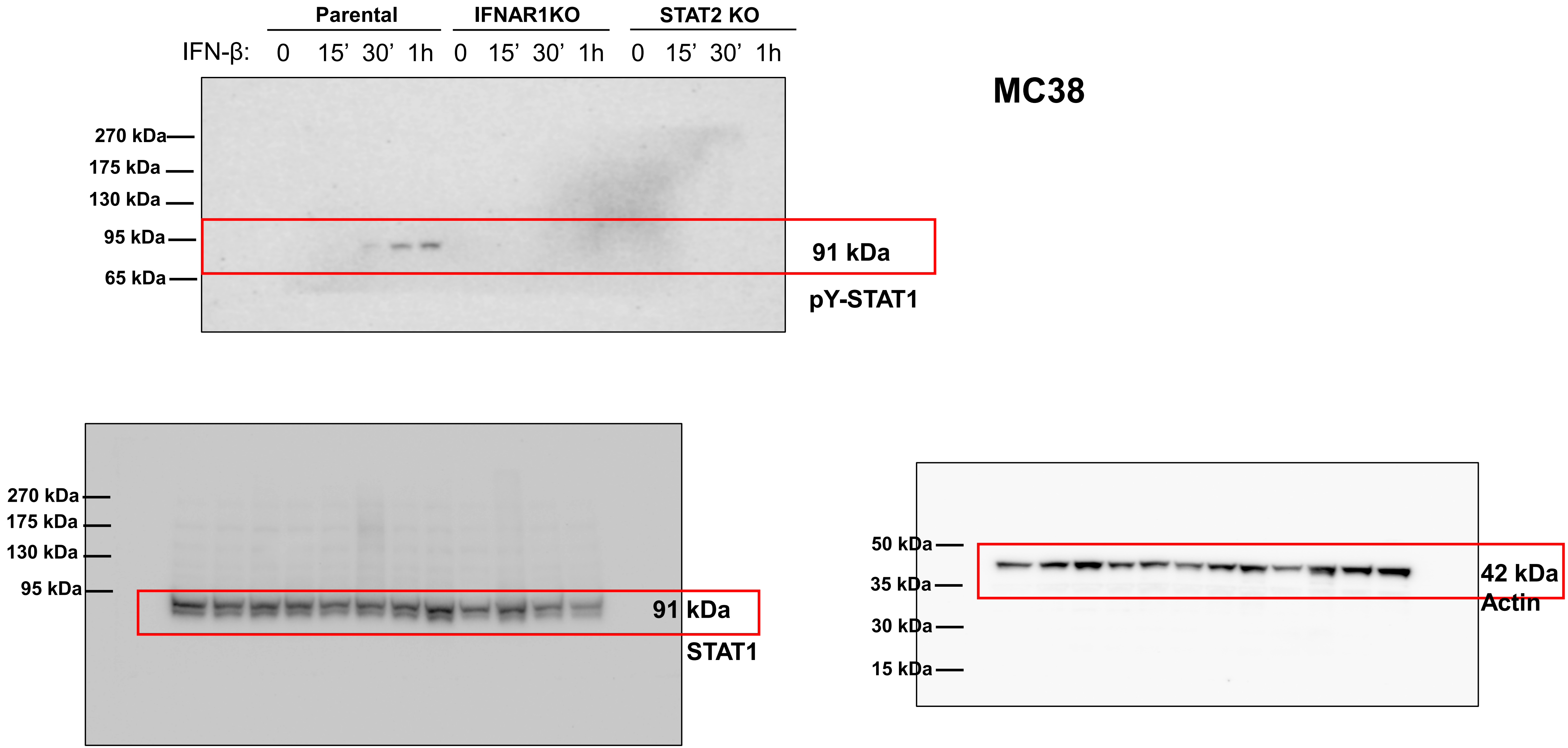

[] : Used in Figure 4a

Figure S4: Activation of STAT3 by IFN-β after deletion of IFNAR1 and STAT2 in MC38 cells

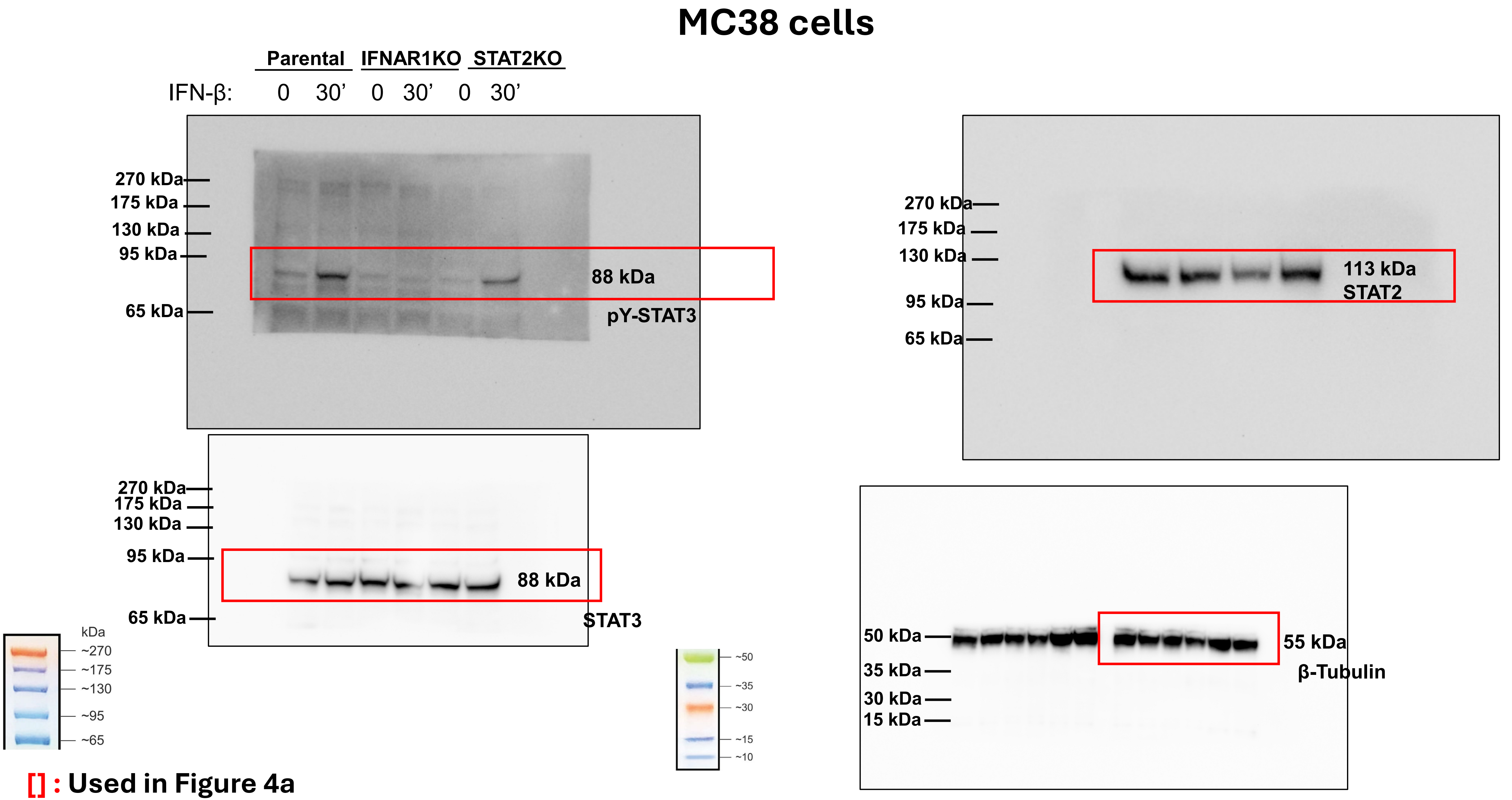

[] : Used in Figure 4a
